# Targeted bisulfite sequencing of the dynamic DNA methylome

**DOI:** 10.1186/s13072-016-0105-1

**Published:** 2016-12-03

**Authors:** Michael J. Ziller, Elena K. Stamenova, Hongcang Gu, Andreas Gnirke, Alexander Meissner

**Affiliations:** 1 Max Planck Institute of Psychiatry, 80804 Munich, Germany; 2 Broad Institute of MIT and Harvard, Cambridge, MA 02142 USA; 3 Harvard Stem Cell Institute, Cambridge, MA 02138 USA; 4 Department of Stem Cell and Regenerative Biology, Harvard University, Cambridge, MA 02138 USA; 5 Max Planck Institute for Molecular Genetics, 14195 Berlin, Germany

**Keywords:** DNA methylation, Bisulfite sequencing, Target enrichment

## Abstract

**Background:**

The ability to measure DNA methylation precisely and efficiently continues to drive our understanding of this modification in development and disease. Whole genome bisulfite sequencing has the advantage of theoretically capturing all cytosines in the genome at single-nucleotide resolution, but it has a number of significant practical drawbacks that become amplified with increasing sample numbers. All other technologies capture only a fraction of the cytosines that show dynamic regulation across cell and tissue types.

**Results:**

Here, we present a novel hybrid selection design focusing on loci with dynamic methylation that captures a large number of differentially methylated gene-regulatory elements. We benchmarked this assay against matched whole genome data and profiled 25 human tissue samples to explore its ability to detect differentially methylated regions.

**Conclusions:**

Our target capture design fills a major gap left by all other assays that exist to map DNA methylation. It maintains the ability to link cytosine methylation to genetic differences, the single-base resolution and the analysis of neighboring cytosines while notably reducing the cost per sample by focusing the sequencing effort on the most informative and relevant regions of the genome.

**Electronic supplementary material:**

The online version of this article (doi:10.1186/s13072-016-0105-1) contains supplementary material, which is available to authorized users.

## Background

DNA methylation, most commonly at cytosines in the CpG dinucleotide, plays an important role in gene and genome regulation [1, 2]. Despite decades of elegant work, we continue to learn more about how and in what context DNA methylation functions through an ever-increasing collection of data. New insights are frequently enabled by technical advances in our ability to effectively map and quantify DNA methylation [3]. While numerous technologies exist, the most widespread are all based on the principle of sodium bisulfite-induced selective deamination of unmethylated cytosine to uracil [4] with either microarrays or sequencing as read-out. Whole genome bisulfite sequencing (WGBS) has the advantage of theoretically capturing all cytosines in the genome at single-nucleotide resolution [5], but it has also a number of practical drawbacks that become amplified with increasing sample numbers. These include the sequencing cost to achieve sufficient coverage, data storage and computing time as well as the fact that most of the genome is depleted of CpGs and hence many reads lack any relevant information. Moreover, the majority of CpGs are static and do not change their methylation state across cell and tissue types, decreasing the information content of WGBS reads even further [5].

## Results

**Fig. 1:**
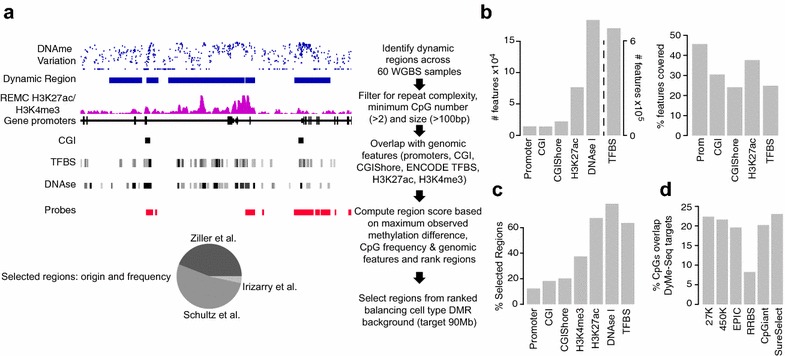
**a** *Top* Genome browser shot illustrating part of the target selection process for the DyMe-Seq assay design. DNAme—DNA methylation; REMC H3K27ac/H3K4me3—union peak set of H3K27ac or H3K4me3 ChIP-Seq data across 70 and 71 distinct cell types each from the Roadmap Epigenome Project; CGI—CpG island; TFBS—transcription factor binding site data across ENCODE transcription factors and cell types; DNAse—Union of DNAse I hypersensitivity sites across 92 cell types form the ENCODE project; Probes—footprint of the final hybrid capture probe set. *Right* Flowchart of filters and prioritization steps during the target selection process. *Bottom* Origin of regions selected for the DyMe-Seq targets by dataset. **b** Total number (*y*-axis, *left chart*) or percentage (*y*-axis, *right chart*) of distinct genomic features (*x*-axis) theoretically covered by the DyMe-Seq assay. Prom—promoter, CGI—CpG island, CGIShore—CpG island shore, H3K27ac—union of all H3K27ac peaks identified in 70 distinct cell types from the Epigenome Roadmap project, DNAase I—DNAse I hypersensitive clusters across ENCODE cell types, TFBS—transcription factor binding sites identified across all ENCODE TF chromatin immunoprecipitation sequencing (ChIP-Seq) experiments. **c** Percentage of regions covered by DyMe-Seq that overlap with different genomic features. Prom—promoter, CGI—CpG island, CGIShore—CpG island shore, H3K27ac—union of all H3K27ac peaks identified in 70 distinct cell types from the Epigenome Roadmap project, H3K4me3—union of all H3K27ac peaks identified in 70/71 distinct cell types from the Epigenome Roadmap project, DNAase I—DNAse I hypersensitive clusters across ENCODE cell types, TFBS—transcription factor binding sites identified across all ENCODE TF chromatin immunoprecipitation sequencing (ChIP-Seq) experiments. **d** CpG level overlap between DyMe-Seq and commonly used array or sequencing based methylation profiling methods. The *y*-axis is the percentage of CpGs targeted by the respective assays that are also part of the by DyMe-Seq target set. 27K, 450K and EPIC—Illumina Methylation bead arrays, RRBS—reduced representation bisulfite sequencing, CpGiant—off-the-shelf SeqCap Epi targeted bisulfite sequencing assay from Roche-NimbleGen. SureSelect—off-the-shelf targeted bisulfite-sequencing assay from Agilent

To overcome the limitations of WGBS while retaining its advantages of single-base resolution, the ability to compare neighboring CpGs on the same read, assign single nucleotide polymorphisms (SNPs) and cover the most relevant parts of the genome, we developed a cost-effective targeted bisulfite sequencing assay for the Dynamic Methylome (DyMe-Seq) that covers CpGs known to change their methylation state across cell and tissue types (see “Methods” section). To this end, we first compiled a list of loci that is highly enriched for dynamic CpGs and includes a large number of gene-regulatory elements and then implemented a hybrid-selection-based targeted bisulfite sequencing strategy for this prime subset of the human methylome—akin to the exome for human genome sequencing. Targeted bisulfite sequencing can be performed either by bisulfite conversion of hybrid-selected native DNA [6] or by hybrid selection of converted DNA [7, 8]. We adopted the latter approach which is commercially available as SeqCap Epi (Roche), trading the superior target specificity of native hybrid selection for lower DNA-input requirements and the ability to capture both strands of bisulfite-converted genomic DNA to distinguish a C to U bisulfite conversion from a C to T SNP. Targeted bisulfite sequencing by post-conversion SeqCap Epi capture has been technically validated previously and displays no apparent capture bias due to DNA methylation states [7, 8]. In contrast to prior designs, we took full advantage of an unprecedented amount of multilayered data to choose the most informative sequencing targets. As outlined in Fig. 1a, we utilized a well-curated set of genome-wide data for DNA methylation (*n* = 60 WGBS datasets) and extensive chromatin maps (H3K4me3 and H3K27ac) from the Roadmap Epigenome Project [9], DNAse I hypersensitive sites in 92 cell types and tissues, and transcription factor (TF) binding data (*n* = 165 TFs across 10 cell and tissue types) from ENCODE [10] and our previous work [11].

The triage process for differentially methylated regions (DMRs) and dynamic CpGs from the two main data sources [5, 12] is outlined in Additional file 1: Figure S1a-d. Our final list of 119,809 DyMe-Seq targets encompasses 91,039,504 bp of which approximately 90 Mb harboring 2.3 M million CpGs constitute legitimate capture space of the corresponding SeqCap Epi probes (Additional file 2). The chosen ~3% of the genome does not comprise all targetable dynamically methylated loci. Nor is 90 Mb the upper limit of the targeting technology. Rather, our curated DyMe-Seq target list represents a carefully balanced compromise between information content, number of capture probes as well as sequencing cost per sample. By design, our set of targets is predominantly enriched for differentially methylated putative regulatory regions and TF binding sites (TFBS) with >90% carrying H3K27ac or H3K4me3 annotations, while maintaining a representative coverage of classic genomic features such as promoters, CpG Islands (CGIs) and CGI shores (Fig. 1b,c). Importantly, our target set captures on average 44% of all putative enhancer regions that are enriched for H3K27ac chromatin marks across 87 distinct cell types or tissues (Additional file 1: Figure S1e). Our final list of prime targets still includes ~40% of our unranked initial list of candidate dynamic CpGs that were filtered solely by repeat content (≤60%), number of CpGs (≥2) and length (≥100 bp) of target regions, irrespective of overlap with genomic features (Additional file 1: Figure S1d).

The gold standard in the field remains WGBS, but as noted its cost and inefficiency are limiting its broad use despite decreasing sequencing costs. The advantage of sequencing only 90 Mb of highly informative targets instead of WGBS becomes clear when comparing the reads required for each sample to achieve 30× coverage across an increasing number of samples: 74 million versus 1.7 billion 100-base reads for 100 samples, covering each CpG in at least 80% of all samples (Additional file 1: Figure S1f). As would be expected, our design always captures a fraction of CpGs that are also covered by other common platforms including methylation bead arrays (Illumina), reduced representation bisulfite sequencing [13] and off-the-shelf targeted bisulfite-sequencing assays (Roche, Agilent). However, the overlap with any single one is less than 25% (Fig. 1d). Hence, DyMe-Seq fills an important gap between existing targeted assays and comprehensive WGBS (Additional file 1: Figure S1g, h).

Next, we assessed key performance properties of DyMe-Seq including genomic biases, coverage distribution, input requirements, off-target and PCR-duplicate rates (Additional file 3: Figure S2). This analysis revealed minimal differences in GC content distribution of the captured targets among technical replicates (Additional file 3: Figure S2b). Lowering the amount of input DNA increased the PCR-duplication rates from 3% for 1 microgram to 6% and 15% for 500 and 250 ng, respectively (Additional file 3: Figure S2c), when using a conventional library preparation strategy where DNA fragments are ligated to sequencing adapters prior to bisulfite conversion (“Methods” section). This result prompted us to continue with an input amount of 500 ng for most subsequent analysis. However, we also evaluated the possibility of lowering the input amounts further by switching to a “bisulfite-first” pre-capture library protocol (Swift Biosystems), yielding dramatically lower PCR-duplication rates essentially constant across an input range from 500 ng down to 160 ng (Additional file 3: Figure S2d). Finally, evaluation of our off-target rate revealed that on average 30% of all reads map outside of the targeted regions (Additional file 3: Figure S2e, f). Based on these analyses, we conclude that 30× coverage requires only 48 million reads for a single library (assuming a 10% duplication rate and 30% off-target reads). However, as noted above, comparing 100 samples where each CpG is covered 30× in at least 80 samples will require more reads (~74 M) per library.

**Fig. 2:**
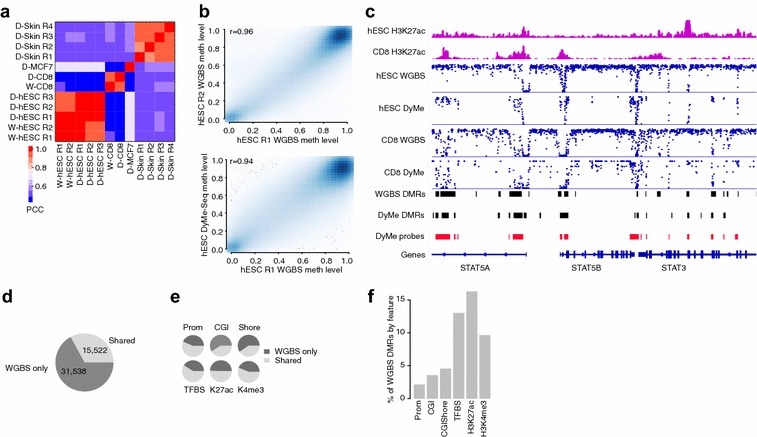
**a** Pearson correlation coefficient (PCC) of three distinct cell types profiled by whole genome bisulfite sequencing (WGBS, W-) and/or DyMe-Seq (D-). The PCC was calculated across all 200-bp tiles of the target region set, excluding all tiles not covered by at least 10 reads in all samples. **b** Scatter plot of CpG methylation levels across 200-bp tiles of the DyMe-Seq target set for two replicates of human embryonic stem cells (hESCs), each profiled with more than 1 billion reads (*left*) and one hESC WGBS replicate (*x*-axis) and an independent replicate of hESCs profiled by DyMe-Seq (*y*-axis) using approx. 40 million high-quality aligned reads. *R* indicates the Pearson correlation coefficient between the two samples. **c** IGV screenshot for the STAT5 locus (chr17:40,397,503-40,503,980), a key gene involved in T-cell function. From *top* to *bottom*: H3K27ac distribution in hESCs and CD8 T cells, methylation level distribution of all covered CpGs for hESCs profiled by WGBS and DyMe-Seq as well as CD8 T cells profiled by WGBS and DyMe-Seq. Each *blue dot* represents a single CpG, and the position on the *y*-axis indicates its methylation level (scale 0–100%). *Black bars* represent DMRs identified between hESCs and CD8 based on the WGBS or DyMe-Seq using one replicate each. DMRs are defined as tiles of our target region set that exhibit a significant methylation difference (*q* value ≤0.05, BH-corrected Fisher’s exact test *p* value) exceeding 20%. Dynamically methylated tiles within 200 bp of each other were then merged into larger DMRs. *Red bars* indicate positions of DyMe-Seq targets across this locus. **d** Pie chart indicating the number of DMRs identified between hESCs and CD8 in the WGBS data that were not recovered by DyMe-Seq (WGBS only) or found with both (Shared). **e** Percentage of WGBS defined hESC-CD8 DMRs overlapping with selected genomic features. **f** Pie charts showing the fraction of WGBS hESC-CD8 feature DMRs that are recovered in the WGBS only or found in both WGBS and DyMe-Seq

Next, we compared DyMe-Seq to WGBS on a number of matched samples. We used high-coverage WGBS samples (1.2–1.5 billion reads) from CD8 positive primary T cells and human embryonic stem cells (hESCs) as a reference and generated between 41 and 129 million targeted DyMe-Seq reads from each cell type. This comparison revealed high correlation of methylation levels of 200-bp tiles across the genome among DyMe-Seq technical replicates (*r* = 0.9 for *n* = 4 and *r* = 0.96 for *n* = 3 distinct sets of technical replicates, Fig. 2a) that is comparable to correlation levels between biological replicates generated by WGBS (Fig. 2a, b). The power to discriminate between cell types is not different from WGBS based on CD8 versus hESC comparison (Fig. 2a). Most importantly, correlation between WGBS and DyMe-Seq on independent biological replicates generated years apart is also very high (*r* = 0.94, Fig. 2b). Subsequently, we confirmed that DyMe-Seq does not exhibit capture biases based on CpG methylation status. To that end, we compared the distribution of methylation level differences as a function of WGBS based methylation levels between two biological replicates of WGBS data and one WGBS and DyMe-Seq sample from the same cell type. This analysis revealed no difference between WGBS and DyMe-Seq (Additional file 3: Figure S2g). The browser shot provides a representative example of WGBS and DyMe-Seq data for a specific locus (Fig. 2c). Our assay covers more than one quarter of DMRs detectable by WGBS (Fig. 2d) and captures the majority of the more meaningful DMRs that directly overlap with known regulatory features (Fig. 2e, f), while sequencing only 3% of the genome. We also note a global change in methylation levels from hESC (cell culture, 74% global methylation) to primary CD8 cells (62% global methylation).

**Fig. 3:**
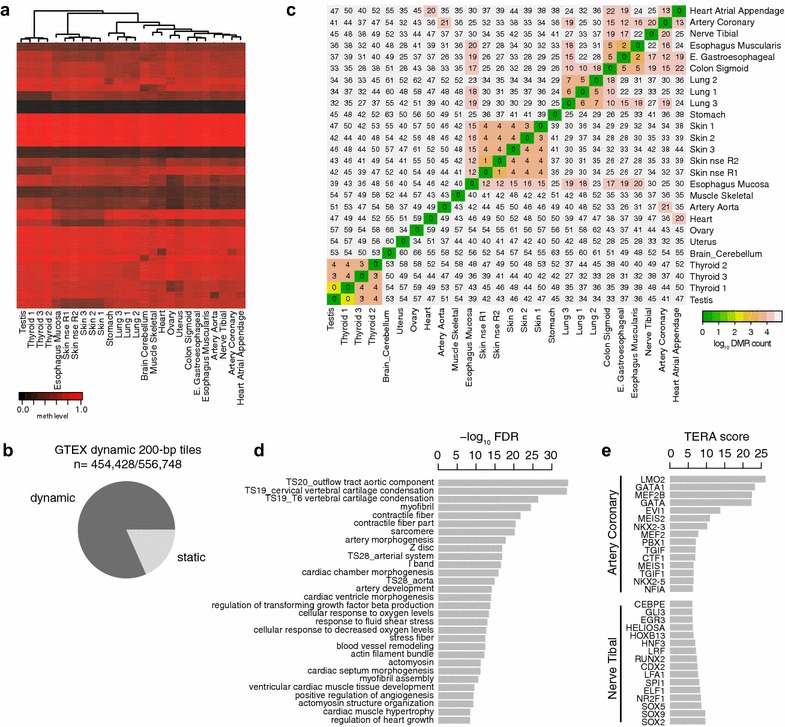
**a** Representative subset of differentially methylated 200-bp tiles across the 26 DyMe-Seq datasets (*N* = 50,000, randomly selected) generated from the entire GTEx pilot cohort (*n* = 25). Skin nse (not sun exposed) 1 and 2 are technical DyMe-Seq replicates from the same DNA samples. Thyroid 1–3, skin 1–3 and lung 1–3 are samples from three different individuals. *Each row* represents one 200-bp tile, and the *color* indicates the methylation level (*black* unmethylated, *red* highly methylated). **b** Fraction of differentially methylated (dynamic) and static 200-bp tiles across the GTEx cohort. A tile was called differentially methylated if the methylation difference exceeded 0.3 at a *q* value ≤0.05 (Fisher’s exact test, BH-corrected) between any of the samples. **c** Overview of number of DMRs across all pairwise comparisons within the GTEx cohort. Here, differentially methylated tiles within 400 bp of each other were merged into DMRs. **d** Gene set enrichment analysis for comparatively hypomethylated regions between the GTEx sample coronary aorta and nerve tibial. Shown are selected gene set categories from the top 40 significantly enriched gene sets (see “Methods” section for details). **e** Top 15 transcription factor motifs enriched in regions differentially methylated between coronary aorta and nerve tibial based on the transcription factor epigenetic remodeling activity (TERA) framework using the a de-methylation score. Transcription factors with high scores are likely to bind frequently to comparatively hypomethylated regions in the respective cell type

To further explore the utility of our assay, we profiled 25 human samples representing 18 different tissues from the genotype-tissue expression (GTEx) cohort [14] (Additional file 4: Figure S3a) and found reliable quantification and identification of DMRs, recovering cell type and inter-individual variation (Fig. 3a). Notably, and in contrast to WGBS data, a large fraction of the sequencing data (82% of 200 bp tiles with DyMe-Seq data) is informative and displays significant methylation differences (Fig. 3b). A subset remains static as would be expected since the 25 samples did not cover all possible cell and tissue types. Adding for instance hESC data (not part of GTEx) will increase the proportion of dynamic 200-bp tiles by another ~2.8% (Additional file 4: Figure S3b). We find a very high correlation between technical replicates (Skin nse [not sun exposed] R1 and R2) and also note the capacity to detect inter-individual variation (Fig. 3c; see lung and skin samples). Lastly, DyMe-Seq captures a representative fraction of the biologically relevant DMR features: Gene set enrichment analysis of DMRs between GTEx heart and nerve tissue reveals a strong enrichment with a number of key pathways of heart and muscle development and function (Fig. 3d). Furthermore, analysis of TFBS [15] within these DMRs identifies key heart (NKX, LMO, GATA) and neural TFs (SOX, GLI, FOXA), suggesting that our assay captures (by design) a representative fraction of the tissue-specific TFBS repertoire (Fig. 3e).

## Discussion

To meet the continuously growing need for more efficient ways to capture DNA methylation information, we designed a hybrid selection target set for a carefully curated list of the most dynamically regulated CpGs (the set also captures 20.8 million CpAs, the predominant non-CpG methylation sites in selected cell types) and demonstrate its application across 20 distinct cell and tissue types. Our approach is substantially more economical than WGBS in terms of sequencing cost per sample, while providing higher coverage of many of the same, relevant regions. The savings in sequencing costs will often far outweigh the cost of performing the additional enrichment step which can be multiplexed by pooling barcoded libraries prior to hybrid selection [8]. However, the choice of the appropriate DNAme profiling assay will ultimately depend on the specific scientific question. If it is critical to detect as many DMRs as possible, high-coverage WGBS may be the only feasible solution.

## Conclusions

Our cost-effective DyMe-Seq assay will be a valuable tool not only for the methylation and developmental biology community, but specifically for many areas of clinical research, including prognostic and biomarker discovery as well as the emerging field of epigenome wide association studies (EWAS) [16].

## Methods

### Identification of target set

We considered two main sources of DMRs: those identified in Ziller et al. 2013 (minus those hypomethylated in sperm) [5] and those defined in Schultz et al. [12] using the same analytical strategy. We then filtered the Schultz et al. list for those already found in Ziller et al. 2013 and used these two sets as our starting DMR set. In addition, we included a subset of tissue specific DMRs (T-DMRs) that were not included in the aforementioned sets [17]. We then proceeded with the analysis of these DMR sets separately for several steps, excluding the sex chromosomes from our analysis. In particular, we filtered these lists stringently in order to identify regions suitable for a hybrid capture approach. To that end, we only included DMRs with a repeat content below 60% based on the repeat masker annotation and at least two dynamic CpGs based on a minimum observed methylation difference of at least 30% between any two samples included in each DMR set. Subsequently, we computed a region-level score based on: 1. the density of dynamic CpGs, 2. the maximum observed methylation difference, 3. the overlap with a putative enhancer regions, defined as the union peak set of H3K27ac peaks across 70 distinct cell types from the ENCODE and Epigenome Roadmap Project, or an annotated RefSeq promoter (±1 kb of TSS). These three scores were each rescaled to the unit interval and then simply added for each DMR. We then ordered the DMRs within each set according to this score. In order to avoid that DMRs originating from one cell type dominate the set of selected regions, we next determined for each DMR the sample with its minimum methylation level. We then selected 53 Mb of DMRs from the Ziller et al. set according to the previously computed score, but maintaining a balanced representation of DMRs originating from all 23 cell types in the collection. This effectively skips regions with higher scores, if their most hypomethylated sample condition was already higher represented in the selected capture set than other samples. Since one purpose of this DMR set is the application to cohorts of many individual such as GTEx, we also selected all DMRs from the remainder of the ranked list that overlapped with eQTLs identified by the GTEx consortium [14] in the following tissues: blood, lung, subcutaneous adipose tissue, skeletal muscle and stomach. In a similar fashion, we selected 30 Mb from Schultz et al. DMR set and identified a 30-Mb set of DMRs. Finally, we also included a set of DMRs that were identified with the Illumina methylation 450 K array across multiple tissues, exhibiting a methylation difference of at least 30% and were not yet present in our final selected DMR set. Together, the union of these DMR lists constituted our initial candidate region set for the DyMe-Seq assay, comprising a total of 95 Mb. Next, this set was further optimized and filtered by Roche-NimbleGen in order to remove regions not suitable for capture, leaving us with 91,039,504 bp and 119,809 target regions (Additional file 2).

### Data processing

Raw reads were aligned to the human genome (hg19) using bsMap 2.7 [18] with the following parameters bsmap -v 0.1 -s 16 -q 20 -w 100 -S 1 -u –R. Subsequently, we used picard tools (http://picard.sourceforge.net) version 1.139 to further process and QC the aligned data files. In particular, we used MarkDuplicates with standard parameter settings to mark and remove likely PCR duplicates, CollectAlignmentMetrics to compute basic alignment statistics, and CalculateHsMetrics with Additional file 2 to calculate all hybrid capture-related metrics, including the on target rate. In order to determine the methylation state of all CpGs captured and assess the bisulfite conversion rate, we used the mcall module in the MOABS [19] software suite with standard parameter settings. Finally, we converted the resulting CpG level files to bigBed files for visualization in the IGV [20], filtering out all CpGs that were covered with less than five reads.

### Data analysis

Analysis of DyMe-Seq data was conducted in R using the methylKit [21] package and a 200-bp tiling of the target capture set. To that end, we imported the CpG level methylation call files from mcall into R using the methylKit function *read* and then computed the weighted methylation mean across for each 200-bp tile using the function *getData*, weighting the methylation level of each CpG with its coverage. We then merged the tile level methylation information across all samples and retained only those tiles covered with more than 10 reads in 70% or more of all samples. To compute differentially methylated tiles, we deliberately decided to choose a simple approach and performed Fisher’s exact test on all pairs of samples for each tile (see Additional file 5: R script). Subsequently, we corrected the resulting p-values using Benjamini–Hochberg [22] correction and defined regions with a *q* value ≤0.05 and an absolute methylation difference ≥0.3 as differentially methylated. Finally, we merged differentially methylated tiles between two samples into larger DMRs if they were less than 400 bp apart. The results of this analysis are displayed in Fig. 3c.

### Feature annotation

For all genomic features considered in this study, we defined them as overlapping with any of our regions if there was at least 1 shared base. Promoters were defined as RefSeq gene transcription start sites ±1 kb. CpG islands and CpG island shores were defined as previously [5] using the CpG island hunter. H3K27ac and H3K4me3 peak sets were defined as previously described [15]. Briefly, IDR [23] peaks were identified for each cell or tissue type from the ENCODE [10] core cell lines and Roadmap Epigenome Project [9] using the IDR framework. The resulting peak sets were then merged, taking the union of all peaks and defined as the H3K27ac and H3K4me3 reference set. For the transcription factor union set, we used the transcription factor binding site cluster track provided by the ENCODE consortium [10].

### Pathway and TERA analysis

Differentially methylated regions identified using the simple strategy outlined above between the GTEX samples artery coronary and nerve tibial were used an example for pathway and transcription factor binding analysis. For the pathway analysis, we selected all regions hypomethylated (according to the criteria listed above, see Additional file 6: Table S2) and used the web-tool GREAT to identify associated biological themes. The results were filtered according to the GREAT [24] standard criteria and a subset plotted in Fig. 3d. For the transcription factor binding site analysis, we used the ERA approach [15] to determine transcription factor motifs that are associated with differential methylation of 200-bp tiles of differentially methylated target sites across the entire GTEX. We then compute the differential ERA scores between the artery coronary and nerve tibial samples and plot the top 15 motifs associated with each condition in Fig. 3e. In the figure, we replaced the motif name with one representative transcription factors associated with each motif.

### Comparison of WGBS and DyMe-Seq

In order to compare the consistency of WGBS and our approach, we selected two cell types for which high-quality WGBS data were available, HUES64 embryonic stem cells and CD8^+^ T cells, and performed DyMe-Seq with our capture set on the same samples. Next, we performed DMR discovery across the entire genome, now using a state-of-the-art beta-binomial model approach implemented in the DSS [25] package. More specifically, we first ran the dmlTest function on the comparison HUES64 versus CD8 for WGBS and DyMe-Seq separately, using one sample per group and smoothing turned on. Subsequently, we identified differentially methylated DMLs using the callDML function with a *p* value threshold of 0.001. Finally, we merged DMLs and identified DMRs using the callDMR function with the following parameters: delta = 0.3, p.threshold = 0.01, minCG = 2, dis.merge = 200 and otherwise default parameters. For the DyMe-Seq comparison, we then only considered those DMRs that were within 300 bp of one of our target regions. The results of this analysis are displayed in Fig. 2d–f.

### Approximation of coverage requirements

To approximate the total per sample coverage required to capture each CpG with 30X, we followed the Lander–Waterman theory [26]. In particular, we computed the required genome coverage to capture each CpG at 30× in 80% of each sample with more than 95% probability using Poisson statistics. Since the capture of any CpG in one particular sample is independent of the capture in a different experiment, we simply computed the probability to capture a CpG with 30 reads in *N* samples as the Nth product of the Poisson cumulative distribution function as a function of the lambda parameter. Subsequently, we determined the lowest lambda parameter for which the CpG was captured with 95% probability in 80% of the N samples and computed this number for *N* = 1–100. The resulting lambda then represents an approximation of the required read coverage after performing all filtering steps. This approximation does not take into account assay-specific biases or enrichment steps and likely represents more an upper bound on the required coverage. We used this modeling approach to estimate the required coverage for both WGBS and DyMe-Seq. For WGBS, we assumed an effective genome size of 2.7 × 10^9^ and for our assay 90 Mb. To incorporate the effects of PCR duplicates, we assumed a duplication rate of 20% for WGBS and 10% for our assay, based on our empirical observations across many samples. For DyMe-Seq, we additionally assumed an off-target rate of 30% based on our empirical observations. The approximated raw coverage per base was then multiplied by the respective target genome sizes, divided by the read length (100 bp) and adjusted for duplicate and off-target effects. The results are then plotted in Additional file 1: Figure S1f.

### Library preparation and sequencing

All human DNA samples used for this study are listed in Additional file 7: Table S3. Hybrid-selected sequencing libraries were prepared using a custom SeqCap probe pool (Roche) essentially following the manufacturer’s SeqCap Epi protocol except that we lowered the amount of input DNA from 1 to 0.5, 0.25 or 0.16 µg per sample and pooled 2–4 PCR-amplified indexed libraries prior to hybrid selection.

Unless otherwise noted in the main text, pre-capture libraries were constructed following the “adapter-ligation-first” protocol of the Kapa Biosystems kit included in the SeqCap Epi reagents from Roche. Briefly, we sheared input genomic DNA (0.25–0.5 µg in 130 µl in Covaris microTUBES) for 3 min on a LE220 sonicator set to duty factor 30%, peak incident power 140 W and 200 cycler per burst. The sheared DNA (mode ~200 bp) was concentrated with 1.7 volumes of Agencourt AMPure XP beads (Beckman Coulter). Beads were resuspended in 70 µl end repair master mix (Kapa Biosystems). End repair, A-tailing, ligation to indexed 5-methyl-C modified adapters, and dual size selection on AMPure beads to narrow the size distribution of the fragment library was performed according to the SeqCap Epi protocol. After EpiTect Fast bisulfite conversion (Qiagen; extending the two 60 °C cycles to 20 min.), the entire eluate from the spin column (20 µl) was PCR-amplified for 12 cycles in 80 µl of HiFi HotStart Uracil+ ReadyMix (Kapa Biosystems). AMPure cleaned-up PCR products were quantified by Qubit (Thermo Fisher).

To lower input DNA amounts, we tested the “bisulfite-conversion first” library construction protocol of the Accel-NGS Methyl-Seq kit (Swift Biosciences) following the kit instructions with the following exceptions: (1) To minimize the off-target sequencing rate, we sheared the input DNA to ~200 bp fragments using the LE220 settings described above instead of shearing to ~400 bp fragments as recommended for Accel-NGS WGBS libraries; (2) we doubled the PCR volume and used 8 PCR cycles for the pre-capture library amplification in 1× HiFi HotStart ReadyMix (Kapa Biosystems).

SeqCap Epi hybridization reactions contained a total of 1 µg of a pool of 2–4 PCR-amplified pre-capture libraries, a total of 1 nmol of 2–4 index-specific blocking oligonucleotides, and the custom SeqCap probe pool designed for the DyMe-Seq targets listed in Additional file 2. After hybridization (typically 70 h), bead capture, low- and high-stringency washes, the entire bead-bound captured material was amplified by 12 cycles of PCR. Hybrid-selected DyMe-Seq libraries were sequenced on an Illumina HiSeq 2500 instrument in fast mode together with a 10% spike-in of a non-indexed PhiX174 library to generate a median of 65 million indexed 100-base purity-filtered paired reads per library. Alignment rates ranged from 90 to 96%. Mean target coverage ranged from 26× to 63×. The bisulfite conversion rate of cytosines in non-CpG context was 99.6% on average. Standard performance metrics for each library are available in Additional file 7: Table S3.

## Additional files

**Additional file 1: Figure S1** **a**. Overlap of differentially methylated CpGs identified in two independent studies based on non-overlapping sample sets. **Figure S1b.** Fraction of differentially methylated (dynamic) CpGs in the two independent studies from **Figure S1a**. **Figure S1c.** Fraction of differentially methylated regions from both studies in **panel a** combined that are considered candidates for a hybrid capture assay, based on length, number of CpGs and repeat content. **Figure S1d.** Fraction of dynamic CpGs initially selected from the candidate set for targeting by hybrid capture based on a CpG-wise scoring approach. **Figure S1e.** Percentage (y-axis) of putative enhancer regions (H3K27ac^+^) across 87 distinct cell and tissue (x-axis) types that are at least partially overlapping with a DyMe-Seq target region. **Figure S1f.** Estimate of total number of reads per sample required (y-axis) to cover each CpG at 30X across 80% of a given number of samples (x-axis) assuming random unbiased sampling[25] and a DyMe-Seq off-target rate of 30%. **Figure S1g.** Total percentage (y-axis) of each genomic feature (x-axis) captured by the Illumina 450K array. **Figure S1h.** Total percentage (y-axis) of each genomic feature (x-axis) captured by RRBS.

**Additional file 2** DyMe-Seq region genomic coordinates.

**Additional file 3: Figure S2** **a.** Distribution of CpG level read coverage (x-axis) across 4 technical replicates of DyMe-Seq at different mean coverage levels indicated (e.g. R1, c=36.2, indicating a mean CpG coverage of 36.2 reads). **Figure S2b.** Distribution of the observed fraction of GC content across 200-bp tiles of the target DyMe-Seq capture set across 4 technical replicates of DyMe-Seq (R1-R4) at different mean coverage levels (see **Figure S2a.**) and the expected percentage based on analysis of target capture set. **Figure S2c.** Percentage of duplicate reads as a function of genomic DNA input in nanogram for DyMe-Seq using a standard “adapter-ligation first” library preparation method (KAPA). Error bars indicate standard error/range based on n≥2. **Figure S2d.** Percentage of duplicate reads as a function of genomic DNA input in nanogram for DyMe-Seq using a “bisulfite first” library preparation protocol (Swift). Error bars indicate standard error/range based on n≥2. **Figure S2e.** On-target rate for three independent DyMe-Seq experiments. On-target rate is defined as on and near (±250) bait bases divided by the number of passing filter bases aligned. **Figure S2f.** On-target rate for all DyMe-Seq experiments conducted in this study, giving a median On-target rate of 70.3%. **Figure S2g.** Left: Distribution of methylation level differences between two biological replicates of hESC WGBS dataset (y-axis) as a function of the methylation level in WGBS replicate R1 (x-axis) across 200-bp tiles of the DyMe-Seq target capture set. Right: This panel depicts the same distribution type of distribution as on the left, but now shows the methylation level differences between WGBS replicate R1 and a DyMe-Seq dataset for a different biological replicate of hESCs, again condition on the methylation level in WGBS hESC replicate 1.

**Additional file 4: Figure S3** **a.** Heatmap and clustering of all GTEx samples based on the Pearson correlation coefficient (PCC) across the methylation levels of the union of all differentially methylated regions between any of the samples. **Figure S3b.** Pie chart showing the number of differentially methylated 200-bp tiles identified across all GTEx samples, those that arise in addition when adding DyMe-Seq data for hESCs (HUES64) and those that still remain static.

**Additional file 5: Table S4** R-script to determine differentially methylated 200-bp tiles between GTEx samples.

**Additional file 6: Table S2** Differentially methylated regions between GTEx samples artery coronary and nerve tibial.

**Additional file 7: Table S3** GTEx sample list and sequencing statistics.

## Abbreviations

DyMe-Seq: dynamic methylation sequencing
WGBS: whole genome bisulfite sequencing
DMR: differentially methylated region
GTEx: genotype-tissue expression
TFBS: transcription factor binding site
