# Supplementary figures and images for: Targeted bisulfite sequencing of the dynamic DNA methylome
Source: Epigenetics Chromatin. 2016 Dec 3;9:55. doi: 10.1186/s13072-016-0105-1 (PMC5135789; doi:10.1186/s13072-016-0105-1)

**a**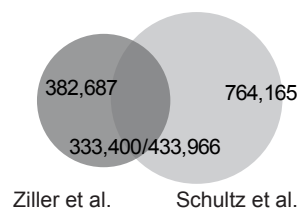**b**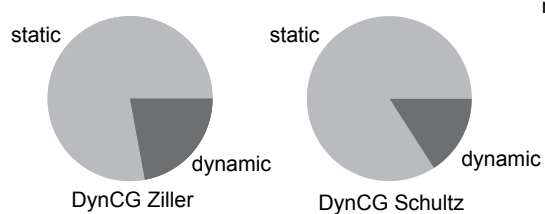**c**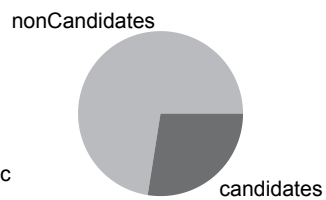**d**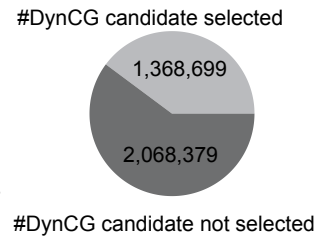**e**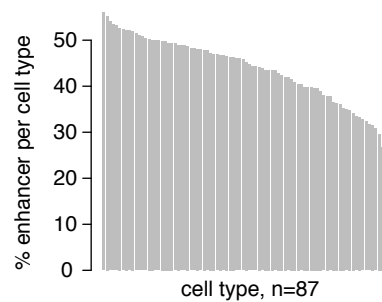**f**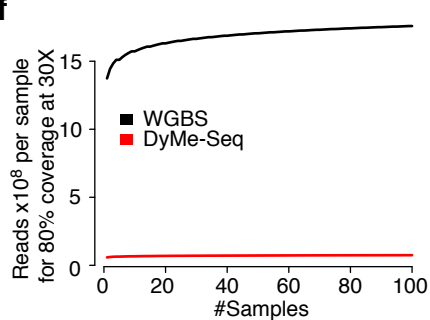**g**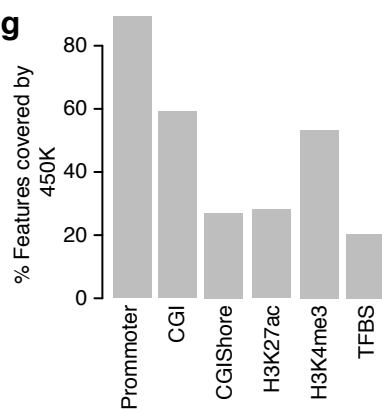**h**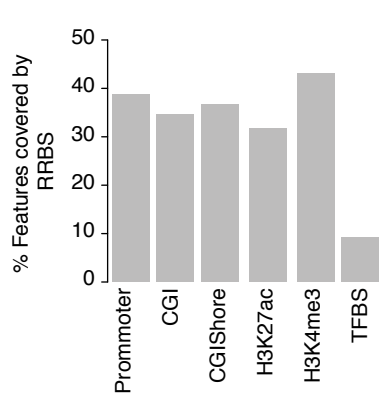

Supplement: Supplementary file 1 — Additional file 1: Figure S1 a. Overlap of differentially methylated CpGs identified in two independent studies based on non-overlapping sample sets. Figure S1b. Fraction of differentially methylated (dynamic) CpGs in the two independent studies from Figure S1a. Figure S1c. Fraction of differentially methylated regions from both studies in panel a combined that are considered candidates for a hybrid capture assay, based on length, number of CpGs and repeat content. Figure S1d. Fraction of dynamic CpGs initially selected from the candidate set for targeting by hybrid capture based on a CpG-wise scoring approach. Figure S1e. Percentage (y-axis) of putative enhancer regions (H3K27ac+) across 87 distinct cell and tissue (x-axis) types that are at least partially overlapping with a DyMe-Seq target region. Figure S1f. Estimate of total number of reads per sample required (y-axis) to cover each CpG at 30X across 80% of a given number of samples (x-axis) assuming random unbiased sampling[25] and a DyMe-Seq off-target rate of 30%. Figure S1g. Total percentage (y-axis) of each genomic feature (x-axis) captured by the Illumina 450K array. Figure S1h. Total percentage (y-axis) of each genomic feature (x-axis) captured by RRBS. [file 13072_2016_105_MOESM1_ESM.pdf]

**a**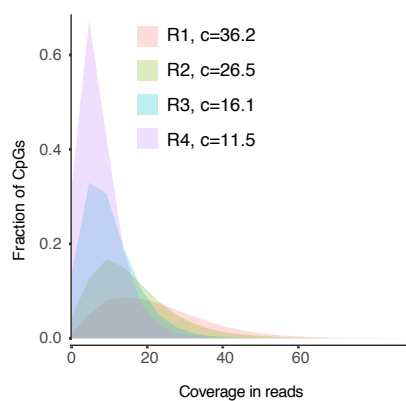**b**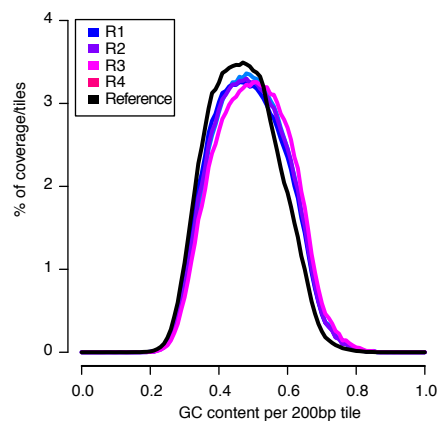**c**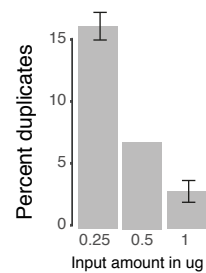**d**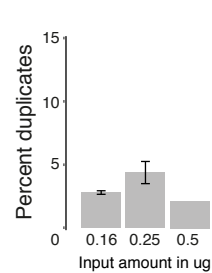**e**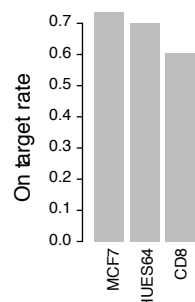**f**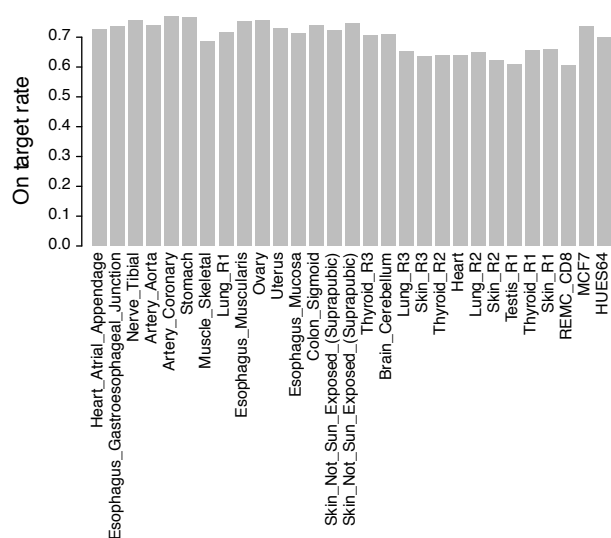**g**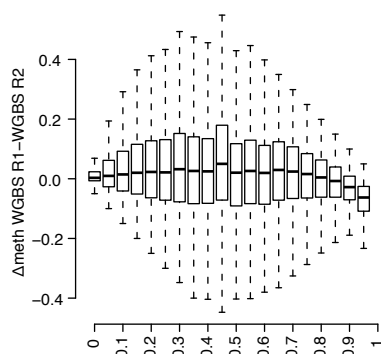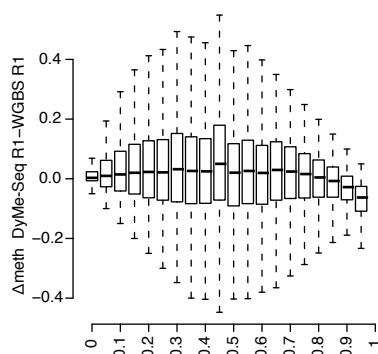

Supplement: Supplementary file 3 — Additional file 3: Figure S2 a. Distribution of CpG level read coverage (x-axis) across 4 technical replicates of DyMe-Seq at different mean coverage levels indicated (e.g. R1, c=36.2, indicating a mean CpG coverage of 36.2 reads). Figure S2b. Distribution of the observed fraction of GC content across 200-bp tiles of the target DyMe-Seq capture set across 4 technical replicates of DyMe-Seq (R1-R4) at different mean coverage levels (see Figure S2a.) and the expected percentage based on analysis of target capture set. Figure S2c. Percentage of duplicate reads as a function of genomic DNA input in nanogram for DyMe-Seq using a standard “adapter-ligation first” library preparation method (KAPA). Error bars indicate standard error/range based on n≥2. Figure S2d. Percentage of duplicate reads as a function of genomic DNA input in nanogram for DyMe-Seq using a “bisulfite first” library preparation protocol (Swift). Error bars indicate standard error/range based on n≥2. Figure S2e. On-target rate for three independent DyMe-Seq experiments. On-target rate is defined as on and near (±250) bait bases divided by the number of passing filter bases aligned. Figure S2f. On-target rate for all DyMe-Seq experiments conducted in this study, giving a median On-target rate of 70.3%. Figure S2g. Left: Distribution of methylation level differences between two biological replicates of hESC WGBS dataset (y-axis) as a function of the methylation level in WGBS replicate R1 (x-axis) across 200-bp tiles of the DyMe-Seq target capture set. Right: This panel depicts the same distribution type of distribution as on the left, but now shows the methylation level differences between WGBS replicate R1 and a DyMe-Seq dataset for a different biological replicate of hESCs, again condition on the methylation level in WGBS hESC replicate 1. [file 13072_2016_105_MOESM3_ESM.pdf]

a

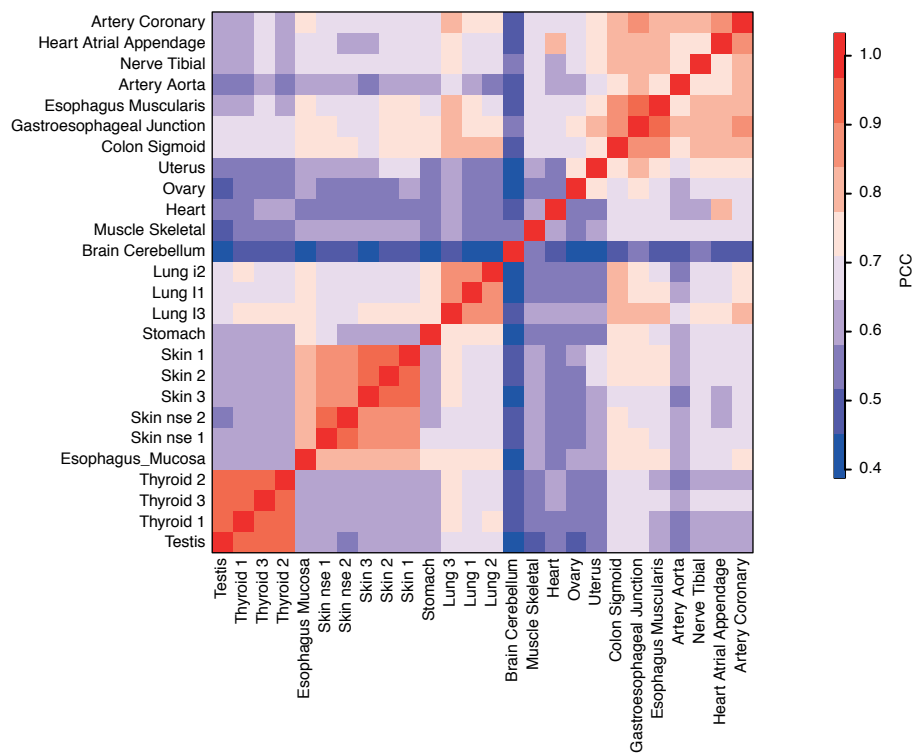

b

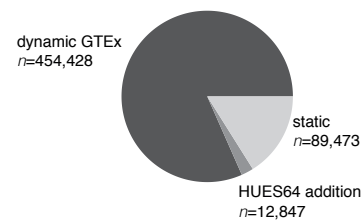

Supplement: Supplementary file 4 — Additional file 4: Figure S3 a. Heatmap and clustering of all GTEx samples based on the Pearson correlation coefficient (PCC) across the methylation levels of the union of all differentially methylated regions between any of the samples. Figure S3b. Pie chart showing the number of differentially methylated 200-bp tiles identified across all GTEx samples, those that arise in addition when adding DyMe-Seq data for hESCs (HUES64) and those that still remain static. [file 13072_2016_105_MOESM4_ESM.pdf]
